# Circulating Serum MicroRNAs as Potential Diagnostic Biomarkers of Posttraumatic Stress Disorder: A Pilot Study

**DOI:** 10.3389/fgene.2019.01042

**Published:** 2019-11-22

**Authors:** Clara Snijders, Julian Krauskopf, Ehsan Pishva, Lars Eijssen, Barbie Machiels, Jos Kleinjans, Gunter Kenis, Daniel van den Hove, Myeong Ok Kim, Marco P. M. Boks, Christiaan H. Vinkers, Eric Vermetten, Elbert Geuze, Bart P. F. Rutten, Laurence de Nijs

**Affiliations:** ^1^Department of Psychiatry and Neuropsychology, School for Mental Health and Neuroscience, Maastricht University, Maastricht, Netherlands; ^2^Department of Toxicogenomics, Maastricht University, Maastricht, Netherlands; ^3^College of Medicine and Health, University of Exeter Medical School, Exeter, United Kingdom; ^4^Department of Bioinformatics (BiGCaT), NUTRIM School of Nutrition and Translational Research in Metabolism, Maastricht University, Maastricht, Netherlands; ^5^Division of Applied Life Science (BK 21), College of Natural Sciences, Gyeongsang National University, Jinju, South Korea; ^6^UMC Utrecht Brain Center, Department of Psychiatry, Utrecht, Netherlands; ^7^Amsterdam UMC (location VUmc), Department of Anatomy and Neurosciences, Amsterdam, Netherlands; ^8^Amsterdam UMC (location VUmc), Department of Psychiatry, Amsterdam, Netherlands; ^9^Arq, Psychotrauma Research Expert Group, Diemen, Netherlands; ^10^Department of Psychiatry, Leiden University Medical Center, Leiden, Netherlands; ^11^Military Mental Healthcare, Netherlands Ministry of Defense, Utrecht, Netherlands; ^12^Department of Psychiatry, New York University School of Medicine, New York, United States

**Keywords:** posttraumatic stress disorder, circulating miRNAs, diagnostic biomarker, trauma, susceptibility

## Abstract

Posttraumatic stress disorder (PTSD) is a psychiatric disorder that can develop upon exposure to a traumatic event. While most people are able to recover promptly, others are at increased risk of developing PTSD. However, the exact underlying biological mechanisms of differential susceptibility are unknown. Identifying biomarkers of PTSD could assist in its diagnosis and facilitate treatment planning. Here, we identified serum microRNAs (miRNAs) of subjects that underwent a traumatic event and aimed to assess their potential to serve as diagnostic biomarkers of PTSD. Next-generation sequencing was performed to examine circulating miRNA profiles of 24 members belonging to the Dutch military cohort Prospective Research in Stress-Related Military Operations (PRISMO). Three groups were selected: “susceptible” subjects who developed PTSD after combat exposure, “resilient” subjects without PTSD, and nonexposed control subjects (*N* = 8 per group). Differential expression analysis revealed 22 differentially expressed miRNAs in PTSD subjects compared to controls and 1 in PTSD subjects compared to resilient individuals (after multiple testing correction and a log2 fold-change cutoff of ≥|1|). Weighted Gene Coexpression Network Analysis (WGCNA) identified a module of coexpressed miRNAs which could distinguish between the three groups. In addition, receiver operating characteristic curve analyses suggest that the miRNAs with the highest module memberships could have a strong diagnostic accuracy as reflected by high areas under the curves. Overall, the results of our pilot study suggest that serum miRNAs could potentially serve as diagnostic biomarkers of PTSD, both individually or grouped within a cluster of coexpressed miRNAs. Larger studies are now needed to validate and build upon these preliminary findings.

## Background

Posttraumatic stress disorder (PTSD) is a psychiatric disorder that can develop upon exposure to a life-threatening traumatic event, i.e., an event capable of producing intense feelings of fear, helplessness, and horror (Association, 2013). Symptoms associated with PTSD include re-experiencing of the traumatic event, avoidance behavior, overall negative mood, and hyperarousal (Association, 2013). The economic burden associated with PTSD is substantial, and patients with PTSD are at increased risk of committing suicide and having familial issues such as marital problems (Fontana and Rosenheck, 1994; Tarrier and Gregg, 2004; Nock and Kessler, 2006; Ferry et al., 2015). Although ∼60% of individuals within Western Europe will one day be exposed to a traumatic event, only ∼6% of these individuals develop PTSD while others show a positive psychological adaptation process denoted as resilience (Kalisch et al., 2017; Koenen et al., 2017). However, some populations such as military soldiers are at elevated risk for trauma exposure, making PTSD a relatively common chronic disorder in the combat Veteran population (Thomas et al., 2017). Currently, a variety of treatment options exist for PTSD, without one being clearly superior to another (Yehuda et al., 2014). Moreover, pharmacological treatment options for PTSD are at best moderately effective and only work for a subset of patients (Richter-Levin et al., 2018). Therefore, increasing efforts are being made to unravel the biological underpinnings of PTSD in order to develop more efficient therapeutic strategies. It is now becoming clear that epigenetic mechanisms are involved in the lasting behavioral and molecular effects of trauma exposure (Schmidt et al., 2011; Snijders et al., 2018a).

Epigenetics refers to a variety of processes that are triggered by environmental factors and cause lasting but reversible alterations in gene expression (Goldberg et al., 2007). Among epigenetic mechanisms, noncoding RNA molecules such as microRNAs (miRNAs) are involved in the posttranscriptional regulation of gene expression by binding to specific messenger RNAs (Peschansky and Wahlestedt, 2014). Several miRNAs have been found implicated in PTSD, shedding much needed light on the underlying pathophysiological underpinnings of this disorder (Wingo et al., 2015; Bam et al., 2016a, Bam et al., 2016b; Martin et al., 2017). Such findings emphasize the notion that expression profiles of miRNAs could one day serve as relatively easily accessible biomarkers or be embedded within a network of several relevant biological processes that together could more accurately reflect the complexity of PTSD. For those individuals who have difficulties recognizing or properly describing their symptoms, identifying such markers could be of use in clinical contexts in order to objectively confirm the presence of the disorder and establish appropriate treatment plans when needed (Lehrner and Yehuda, 2014). Using these markers could be equally relevant during postdeployment medical screenings since military service members may have secondary reasons to not fully disclose their symptoms (Yehuda et al., 2013).

Here, we aimed to identify serum miRNAs that could one day serve as diagnostic biomarkers of PTSD. We further aimed to gain insights in the coexpression patterns of these miRNAs, their predicted gene targets and underlying biological pathways, along with their diagnostic accuracy. We hypothesized that specific miRNAs are differentially expressed between subjects with PTSD, trauma-exposed healthy individuals (referred to as “resilient” subjects in this paper), and nonexposed healthy controls. For this, we performed next-generation sequencing (NGS) on serum samples of 24 military members belonging to a Dutch military cohort, and we compared miRNA profiles between the three groups. Our findings suggest that miRNAs could potentially serve as biomarkers of PTSD, both individually or grouped within a cluster of coexpressed miRNAs. Larger studies are now needed in order to further validate and build upon these preliminary findings.

## Materials and Methods

### Participants

A subset of military personnel (24 males) was selected from the larger Prospective Research in Stress-Related Military Operations (PRISMO) study, a prospective cohort of Dutch military members deployed to Afghanistan for 4 months (Reijnen et al., 2015; Eekhout et al., 2016). Based on the level of combat exposure during deployment and the severity of postdeployment PTSD symptoms, three subgroups were identified: 1) susceptible individuals, i.e., trauma-exposed subjects with deployment-related PTSD symptoms at 6 months follow-up; 2) resilient individuals, i.e., trauma-exposed soldiers with no PTSD diagnosis at follow-up; and 3) controls, i.e., deployed, but nonexposed and mentally healthy military members. Blood samples were collected at the Utrecht University Medical Center at 6 months postdeployment. Trauma exposure was assessed using a 19-item deployment experiences checklist (van Zuiden et al., 2011). The severity of PTSD symptoms was established using the 22-item Self-Rating Inventory for PTSD (SRIP) (Hovens et al., 2002). Information on smoking and alcohol was collected using self-report measures. This study was approved by the ethical committee of University Medical Center Utrecht (01-333/0) and conducted in accordance with the Declaration of Helsinki. All participants gave written informed consent.

### RNA Isolation

Total RNA was isolated from 300μl human serum using the *mir*Vana PARIS kit (Ambion) according to the manufacturer’s instructions. Briefly, the samples were incubated with an equal volume of denaturing solution, acid-phenol/chloroform was added, and the samples spun for 5min at 10,000×*g*. The aqueous phase was recovered and passed through a filter which was washed three times with the provided wash solutions. Final RNA was eluted in 100µl nuclease-free water. The concentrations and quality of the recovered RNA were measured using the Agilent Bioanalyzer 2100 (Agilent Technologies, Inc., CA, USA). All eluates were stored at −80°C until further use.

### Small RNA Library Preparation and Next-Generation Sequencing

Barcoded libraries (*N* = 24, 8 per group) were prepared with an input of 25ng total RNA using the Illumina Small RNA TruSeq kit (Illumina, CA, USA). Briefly, 3′ and 5′ RNA adapters were added, the samples were reverse transcribed and amplified using 11 PCR cycles. All samples were processed in parallel and received a unique barcode. The complementary DNA constructs were gel purified and concentrated by ethanol precipitation. The quality control was performed using Agilent's 2100 Bioanalyzer with a High-Sensitivity DNA Chip. The 24 samples were pooled (*N* = 8 per group) and sequenced in duplicate using the Illumina HiSeq 2000 DNA sequence platform according to the manufacturer’s protocol (GEO accession: GSE137624).

### Small RNA Sequencing Data Analysis

Quality control of the raw sequences was done using FastQC (v. 0.11.3), and reads were preprocessed and mapped to the latest release of miRBase (v. 21) (Baras et al., 2015) utilizing miRge with default settings (Kozomara and Griffiths-Jones, 2014). In order to compensate for bias introduced by very low abundant sequences, only those miRNAs with an average of 50 counts (or more) across samples were considered for further analyses.

### Differential Expression Analysis

Data normalization and differential expression analysis was conducted with the DESeq2 package in R (v. 3.5.2) (Love et al., 2014) thereby correcting for age, alcohol use, and smoking status. Resulting *p*-values were controlled by the false discovery rate (FDR) at 5% (Benjamini and Hochberg, 1995).

### Weighted Gene Coexpression Network Construction and Module Detection

The identified miRNAs were used to construct coexpression networks using the Weighted Gene Coexpression Network Analysis (WGCNA) R package (Langfelder and Horvath, 2008). Normalized miRNA data was used as input. An adjacency matrix was generated by calculating Pearson’s correlations between all miRNAs. Next, topological overlap between miRNAs was calculated using a power of 9. We performed 200 rounds of bootstrapping in order to construct a network that is robust to outliers. The cutreeDynamic function in the dynamicTreeCut R package was then used to identify coexpression modules of positively correlated miRNAs with high topological overlap. Modules with at least 30 miRNAs were assigned a color. Modules with highly correlated eigengenes were merged using the mergeCloseModules function in R. Pearson correlations between module eigengenes, age, smoking status, and alcohol were calculated. Welch’s *t*-tests were performed in order to detect differences between module eigengenes of the control subjects and the trauma-exposed individuals. One-way ANOVAs were performed to detect differences between the three groups. When significant, the post-hoc Tukey HSD test was used to detect pairwise group differences.

### Target Gene Pathway and Enrichment Analyses

The experimentally validated miRNA–target interactions database miRTarBase 6.0 (Chou et al., 2018) was used to identify gene targets of miRNAs. In order to narrow down the amount of target genes for further analyses, one-sided Fisher tests (with FDR multiple correction) were performed to evaluate whether the amount of miRNAs targeting a specific gene was significantly higher than expected by chance. Those genes were then analyzed for enriched Kyoto Encyclopedia of Genes and Genomes (KEGG) pathways and Gene Ontology terms (GO terms) using the online Database for Annotation, Visualization and Integrated Discovery (DAVID) v6.8 (Huang da et al., 2009; Huang da et al., 2009).

### Statistical Analyses

To detect differences in age, number of previous deployments, cigarette smoking, alcohol use, trauma exposure scores, and SRIP scores between the groups, the Welch ANOVA with Games–Howell post-hoc test was applied. Since data on alcohol use at the 6 months follow-up time point was not available for all subjects, predeployment values were used instead. For each individual, smoking status was estimated based on their unique methylation patterns in 183CpGs, as previously described (Zeilinger et al., 2013). Finally, the classification accuracy of specific miRNAs was determined by calculating the area under the receiver operating characteristic (ROC) curve (AUC) in R.

## Results

### Demographic Characteristics

A total of 24 subjects were included in the present study, of which 8 developed PTSD following deployment, 8 were resilient, and 8 were nonexposed controls (**Supplementary Table 1**). Based on the sequencing results, four subjects were excluded due to having a distinctively lower amount of reads causing great variation in expression data between samples. The three groups did not differ in terms of age, number of previous deployments, smoking status, and alcohol use (**Table 1**). On average, subjects with PTSD and resilient individuals were exposed to a similar amount of traumatic events, which was significantly more than the nonexposed controls [*F*(2, 8.8) = 54.67, *p* < 0.001. Games–Howell post-hoc showed *p* < 0.001 for PTSD versus control, and resilient versus control]. Finally, resilient and control subjects had similar postdeployment PTSD scores as measured by the SRIP, which were significantly lower than the average score of the PTSD group [*F*(2, 11.15) = 25.23, *p* < 0.001. Games–Howell post-hoc showed *p* < 0.001 for PTSD versus resilient, and PTSD versus control].

**Table 1:** Demographic characteristics of the 20 subjects remaining after outlier exclusion.

	Susceptible (*N* = 8)	Resilient (*N* = 6)	Control (*N* = 6)	*p*-value
Age when deployed	22.13 (0.61)	34.17 (4.88)	27.50 (3.62)	0.083
Number of previous deployments	0.29 (0.18)	0.83 (0.48)	0.17 (0.17)	0.465
Cigarette smoking	2.79 (1.65)	−1.43 (1.30)	−0.15 (1.89)	0.194
Alcohol use	2.86 (0.67)	1.17 (0.40)	1.83 (0.60)	0.155
Trauma exposure score	7.75 (0.98)	7.17 (0.75)	0.5 (0.22)	<0.001
SRIP PTSD score	55.25 (4.01)	25.50 (1.63)	24.50 (1.46)	<0.001

Data are presented as mean (SE). SRIP, self-rating inventory for posttraumatic stress disorder.

### miRNAs Sequencing and Differential Expression Analysis

**Figure 1:**
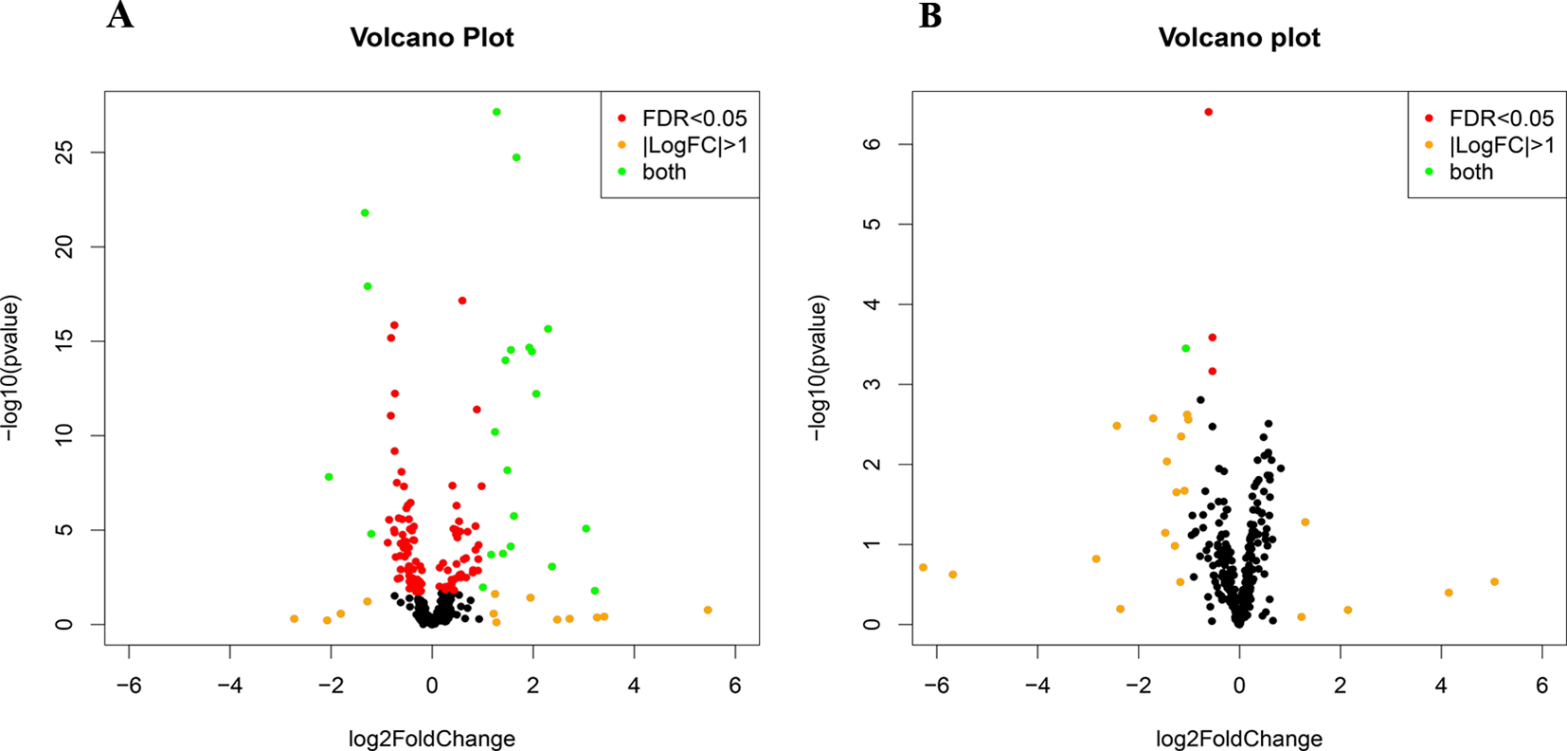
Volcano plots of differentially expressed microRNAs (miRNAs) between posttraumatic stress disorder (PTSD) cases and controls **(A)** and PTSD cases and resilient subjects **(B)**. Black dots represent nonsignificantly differentially expressed miRNAs, red dots represent significant miRNAs with a log2 FC<|1|, orange dots represent nonsignificant miRNAs with a log2 FC≥|1|, and green dots represent significantly differentially expressed miRNAs with a log2 FC≥|1|. Significance is declared when adjusted *p*<0.05.

**Figure 2:**
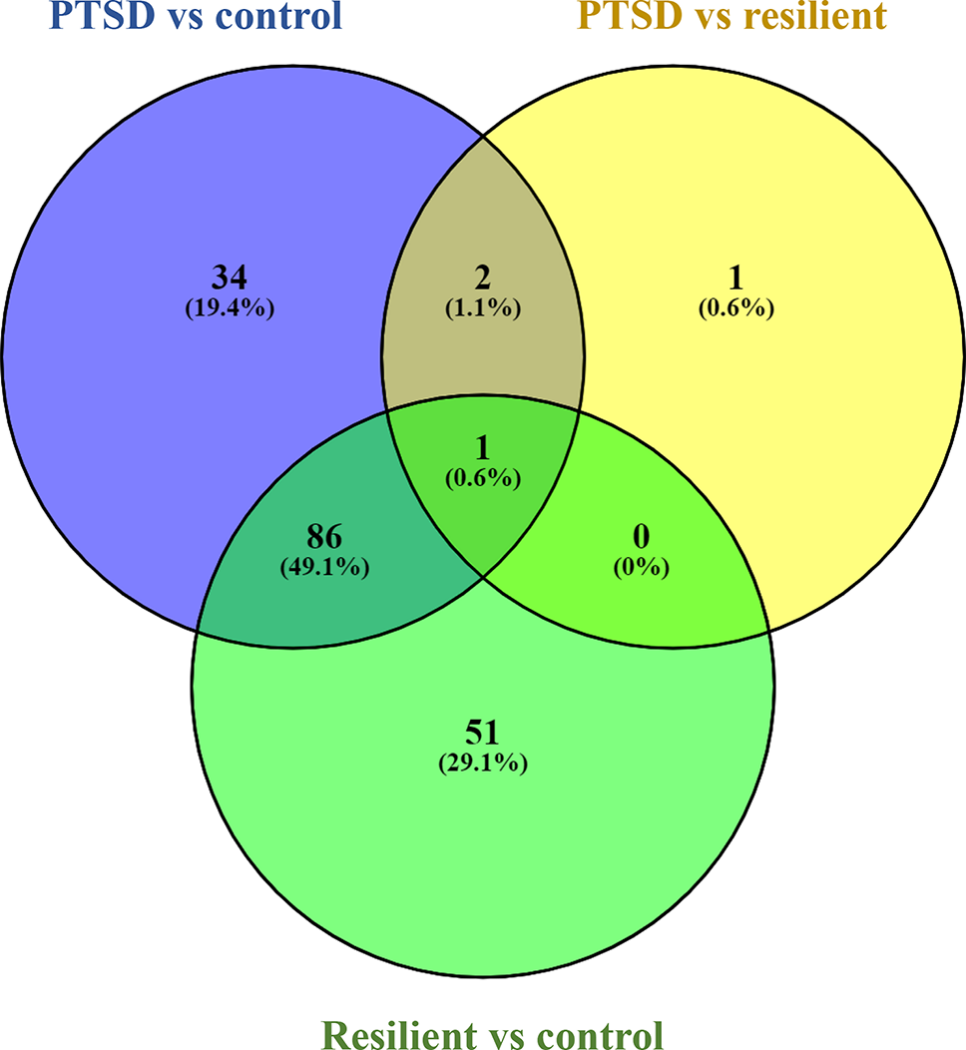
Venn diagram showing overlapping microRNAs (miRNAs). Of particular interest here are the two miRNAs at the intersection of the blue and yellow circle (but not the green), i.e., miR-4454 and miR-210-3p. Obtained using https://bioinfogp.cnb.csic.es/tools/venny/index.html

Small RNA sequencing yielded an average of 9.5 million unfiltered sequencing reads across all samples. After adaptor trimming and size selection, 1.9 million high-quality reads remained, which were aligned to miRNA sequences from miRBase (release 21). As mentioned earlier, principal component analysis revealed the presence of four outliers, which were excluded from further analysis. The count data were then filtered for miRNAs that showed an average of 50 reads or more across all samples. This resulted in the identification of 306 different miRNAs. Differential expression analysis in DESeq2 revealed that a total of 123 miRNAs showed differential expression between PTSD cases and nonexposed controls, while 4 were downregulated in PTSD cases compared to resilient individuals (**Supplementary Table 2**). Selecting those miRNAs with a log2 fold-change (FC) value≥|1.0| and FDR adjusted *p* < 0.05 revealed that one miRNA, miR-1246, was downregulated in PTSD subjects compared to resilient subjects and 22 were differentially expressed between PTSD subjects and nonexposed controls (**Table 2**, **Figure 1**). Of these, 4 were downregulated and 18 were upregulated. We used the Venn tool to identify those differentially expressed miRNAs that are specific for PTSD only (**Figure 2**). Two miRNAs were identified at the intersection of the blue and yellow circles, i.e., miR-4454 and miR-210-3p. Both miRNAs were significantly downregulated in PTSD subjects compared to resilient subjects and controls and not differentially expressed between resilient subjects and controls, suggesting that these could be more specific to PTSD (**Supplementary Table 2**). However, both miRNAs had log2 FC values of −0.61 and −0.54, which does not pass our threshold of ≥|1.0|.

**Table 2:** Differentially expressed microRNAs (miRNAs) between posttraumatic stress disorder (PTSD) cases versus controls and PTSD cases versus resilient individuals with a log2 fold-change value≥|1.0| and FDR adjusted *p* < 0.05.

Nr	PTSD vs. control				PTSD vs. resilient			
	*miRNA*	*Log2 FC*	p*-value*	*FDR adj* p*-value*	*miRNA*	*Log2 FC*	p-*value*	*FDR adj* p-*value*
1	hsa-miR-218-2-3p	3.22	1.65E−02	4.20E−02	hsa-miR-1246	−1.06	3.54E−04	3.05E−02
2	hsa-miR-3609	3.05	8.22E−06	6.62E−05				
3	hsa-miR-432-5p	2.37	8.44E−04	3.31E−03				
4	hsa-miR-138-5p	2.29	2.18E−16	9.54E−15				
5	hsa-miR-221-5p	2.06	6.11E−13	1.34E−11				
6	hsa-miR-4485-3p	1.98	3.45E−15	9.59E−14				
7	hsa-miR-31-5p	1.92	2.16E−15	7.35E−14				
8	hsa-miR-146b-5p	1.67	1.86E−25	2.85E−23				
9	hsa-miR-5096	1.62	1.81E−06	1.84E−05				
10	hsa-miR-222-3p	1.56	2.81E−15	8.61E−14				
11	hsa-miR-1273g-3p	1.55	7.23E−05	3.75E−04				
12	hsa-miR-302a-5p	1.49	6.61E−09	1.06E−07				
13	hsa-miR-221-3p	1.45	1.03E−14	2.62E−13				
14	hsa-miR-619-5p	1.40	1.74E−04	8.31E−04				
15	hsa-miR-335-5p	1.28	7.18E−28	2.20E−25				
16	hsa-miR-146b-3p	1.25	6.30E−11	1.13E−09				
17	hsa-miR-3175	1.17	1.95E−04	9.20E−04				
18	hsa-miR-3656	1.00	1.07E−02	2.86E−02				
19	hsa-miR-184	−1.20	1.58E−05	1.01E−04				
20	hsa-let-7d-5p	−1.27	1.22E−18	9.33E−17				
21	hsa-miR-98-5p	−1.33	1.57E−22	1.60E−20				
22	hsa-miR-146a-5p	−2.04	1.52E−08	2.22E−07				

The table is organized based on decreasing log2 fold-change values. miRNA, microRNA; log2 FC, log2 fold-change.

### Weighted Gene Coexpression Network Analysis

**Figure 3:**
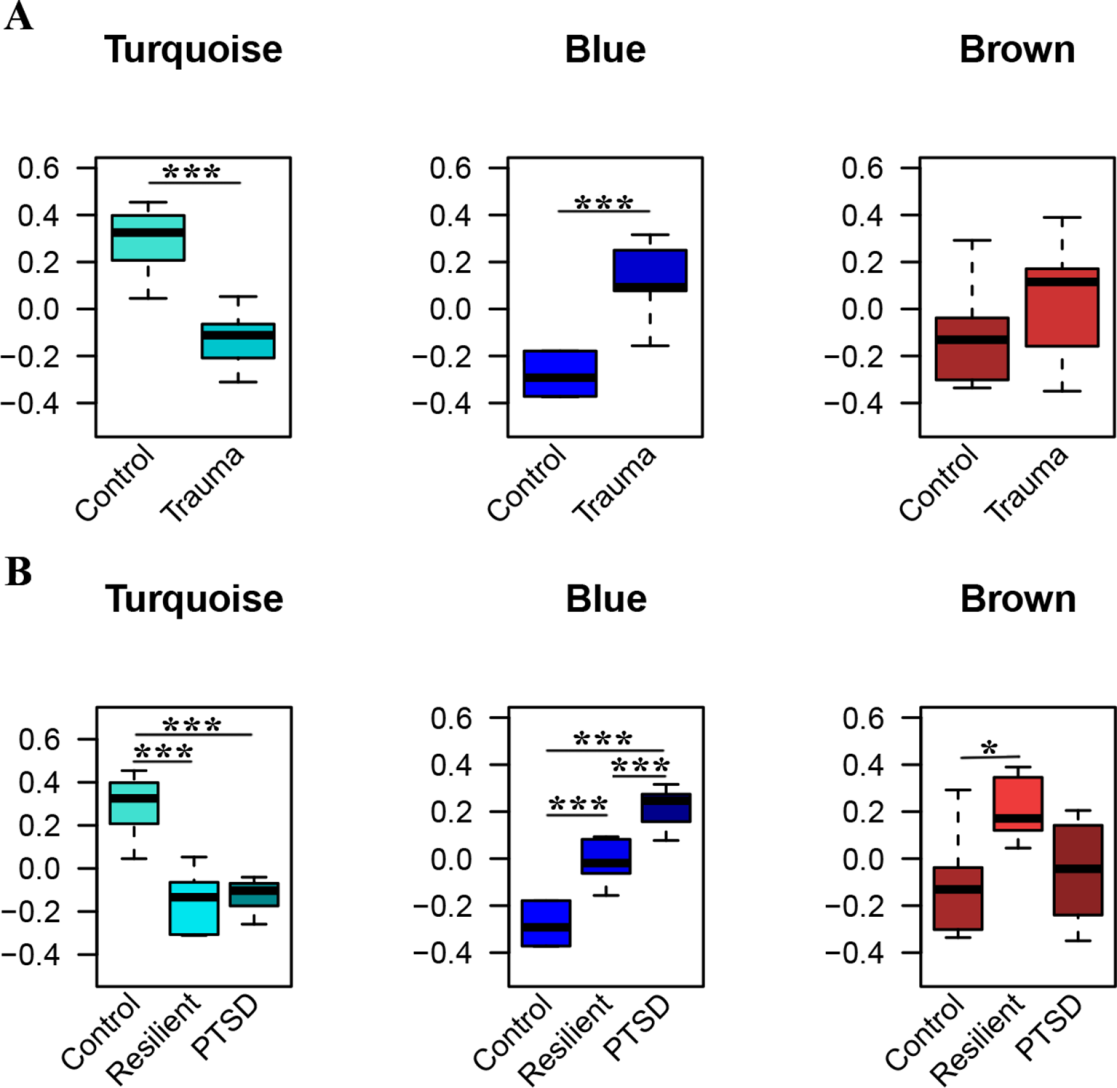
Significant modules of coexpressed miRNAs. The y-axis displays the module eigengene values. Groups are stratified by trauma exposure **(A)**, and trauma exposure and PTSD status **(B)**. Significances were detected using Welch tests **(A)** or one-way ANOVAs **(B)** *p < 0.05, ***p < 0.001.

WGCNA was applied using the 306 identified miRNAs in order to detect clusters of coexpressed miRNAs. Based on the sample dendrogram, one outlier was removed from further analyses (**Supplementary Figure 1**). We identified three miRNA modules (**Figure 3**). The turquoise, blue, and brown modules each had 84, 79, and 65 miRNAs, respectively. None of the modules were associated with the potential covariates age, smoking status, or alcohol use (**Supplementary Figure 2**). Within each module, the module eigengenes were significantly different between trauma-exposed individuals and nonexposed controls for the turquoise and blue modules (*p* = 2.67×10^−04^, *p* = 2.51×10^−06^, respectively) but not for the brown module (*p* = 0.196; **Figure 3A**). When stratifying the trauma-exposed individuals into PTSD subjects and resilient subjects, the individual eigengenes of the blue module were significantly different between PTSD subjects and resilient individuals (*p* = 1.46×10^−03^; **Figure 3B**), which was not the case for the other modules. We therefore focused on the blue module for further analyses.

Out of the 79 miRNAs belonging to this module, 67 were differentially expressed between PTSD subjects and controls (**Table 1**), including miR-138-5p, the hub miRNA (**Table 3**). In order to evaluate the diagnostic accuracy of some of these miRNAs, we performed ROC analysis for those miRNAs with the highest absolute module memberships. The five most contributing miRNAs, i.e., miR-221-3p, miR-335-5p, miR-138-5p, miR-222-3p, and miR-146-5p (**Table 3**), could perfectly distinguish PTSD subjects and controls (AUC of 1 for all miRNAs; **Supplementary Figure 3** A.1 and A.2 for miR-221-3p). These miRNAs could equally well differentiate PTSD subjects from resilient subjects, except for miR-221-3p and miR-222-3p (AUC of 0.95 and 0.98, respectively). When obtaining ROC curves using miRNA expression levels adjusted for confounders (i.e., age, smoking, and alcohol use), all miRNAs could still distinguish PTSD subjects from controls (**Supplementary Figure 3** B.1 and B.2 for miR-221-3p). However, differentiating PTSD from resilience was less accurate as reflected by AUCs of 0.625, 0.775, 0.725, 0.675, and 0.775 for miR-221-3p, miR-335-5p, miR-138-5p, miR-222-3p, and miR-146-5p, respectively (**Supplementary Figure 3** B.1 and B.2 for miR-221-3p).

**Table 3:** MicroRNAs (miRNAs) belonging to the Weighted Gene Coexpression Network Analysis (WGCNA) blue module.

Nr	miRNA	Nr	miRNA
1	hsa-miR-221-3p	41	hsa-miR-641
2	hsa-miR-335-5p	42	hsa-miR-208a-3p
3	hsa-miR-138-5p	43	hsa-miR-18a-3p
4	hsa-miR-222-3p	44	hsa-miR-193a-5p
5	hsa-miR-146b-5p	45	hsa-miR-411-5p*
6	hsa-miR-31-5p	46	hsa-miR-148a-5p
7	hsa-miR-340-5p	47	hsa-miR-505-3p
8	hsa-miR-210-3p	48	hsa-miR-214-3p
9	hsa-miR-208b-3p	49	hsa-miR-335-3p
10	hsa-miR-302a-5p	50	hsa-miR-4485-3p
11	hsa-let-7i-5p	51	hsa-miR-10a-5p
12	hsa-miR-4454	52	hsa-miR-212-3p
13	hsa-miR-146b-3p	53	hsa-miR-331-3p
14	hsa-miR-99b-3p	54	hsa-miR-490-3p*
15	hsa-let-7a-3p	55	hsa-miR-20a-5p
16	hsa-miR-221-5p	56	hsa-miR-455-5p
17	hsa-miR-27a-3p/27b-3p	57	hsa-miR-874-5p
18	hsa-miR-127-3p	58	hsa-miR-675-5p
19	hsa-miR-3200-3p	59	hsa-miR-504-5p
20	hsa-miR-128-3p	60	hsa-miR-654-3p*
21	hsa-miR-20b-5p	61	hsa-miR-30a-3p*
22	hsa-miR-199a-3p	62	hsa-miR-425-3p
23	hsa-miR-181c-5p	63	hsa-miR-183-5p
24	hsa-miR-652-3p	64	hsa-miR-532-3p*
25	hsa-miR-4662a-5p	65	hsa-miR-193b-3p*
26	hsa-miR-17-5p/106a-5p	66	hsa-miR-130a-3p
27	hsa-miR-146a-5p	67	hsa-miR-7151-5p*
28	hsa-miR-148a-3p	68	hsa-miR-23a-3p
29	hsa-miR-708-5p	69	hsa-miR-877-3p*
30	hsa-miR-34a-5p	70	hsa-miR-424-3p
31	hsa-miR-574-3p	71	hsa-miR-151a-3p
32	hsa-miR-145-3p	72	hsa-miR-3175
33	hsa-miR-490-5p	73	hsa-miR-361-3p
34	hsa-miR-148b-5p	74	hsa-miR-92b-5p
35	hsa-miR-143-3p	75	hsa-miR-181a-3p
36	hsa-miR-184	76	hsa-miR-328-3p*
37	hsa-miR-199b-3p	77	hsa-miR-423-3p*
38	hsa-miR-628-5p	78	hsa-miR-139-5p*
39	hsa-miR-132-3p	79	hsa-miR-339-3p*
40	hsa-miR-181c-3p		

miRNAs with a star were not differentially expressed in PTSD cases versus controls or versus resilient subjects. The miRNAs are ranked based on their absolute module membership (highest to lowest).

### Target Gene Pathway and GO Enrichment Analyses

Validated gene targets of the 79 miRNAs comprised within the blue module were obtained from the online database miRTarBase (Chou et al., 2018). In order to narrow down this extensive set of target genes (*N* = 9270), Fisher tests were performed to select only those genes that were targeted by significantly more miRNAs than expected by chance. This revealed a set of 146 genes, which were considered for pathway and enrichment analyses (**Supplementary Table 3**). After FDR adjustment, 15 significantly enriched KEGG pathways were identified of which most were cancer-related (**Table 4**). GO enrichment analyses of these target genes further identified eight significant biological processes, five molecular functions, and six cellular components (**Table 5**). The most enriched GO terms were related to apoptotic processes, protein binding, and intracellular compartments, respectively (**Table 5**).

**Table 4:** Significant Kyoto Encyclopedia of Genes and Genomes (KEGG) pathways.

Nr	Term	*p* value	FDR adj *p* value
1	Pathways in cancer	2.94E−10	3.66E−07
2	Pancreatic cancer	6.27E−09	7.79E−06
3	HTLV-I infection	1.26E−08	1.56E−05
4	Small cell lung cancer	8.93E−08	1.11E−04
5	Melanoma	2.14E−07	2.65E−04
6	Hepatitis B	2.23E−07	2.77E−04
7	FoxO signaling pathway	8.06E−07	1.00E−03
8	Colorectal cancer	9.16E−07	1.14E−03
9	Prostate cancer	1.38E−06	1.71E−03
10	MicroRNAs in cancer	2.34E−06	2.91E−03
11	Chronic myeloid leukemia	2.93E−06	3.63E−03
12	Apoptosis	1.14E−05	1.41E−02
13	Central carbon metabolism in cancer	1.41E−05	1.75E−02
14	MAPK signaling pathway	1.49E−05	1.85E−02
15	Glioma	1.56E−05	1.94E−02

These pathways were identified using the online Database for Annotation, Visualization and Integrated Discovery (DAVID) v6.8.

**Table 5:** Significant gene ontology (GO) terms enriched for a subset of target genes (*N* = 218) of the coexpressed microRNAs (miRNAs) from the blue module (*N* = 70).

Nr	GO ID	GO term	*p*-value	FDR adj *p*-value
	*Biological process*			
1	GO:0008630	Intrinsic apoptotic signaling pathway in response to DNA damage	5.11E−09	8.60E−06
2	GO:0071456	Cellular response to hypoxia	1.19E−07	2.00E−04
3	GO:0071260	Cellular response to mechanical stimulus	1.46E−07	2.45E−04
4	GO:0006919	Activation of cysteine-type endopeptidase activity involved in apoptotic process	4.96E−07	8.35E−04
5	GO:0043066	Negative regulation of apoptotic process	1.54E−06	2.59E−03
6	GO:0045944	Positive regulation of transcription from RNA polymerase II promoter	2.35E−06	3.95E−03
7	GO:0030308	Negative regulation of cell growth	8.64E−06	1.45E−02
8	GO:0097192	Extrinsic apoptotic signaling pathway in absence of ligand	9.20E−06	1.55E−02
	*Molecular function*			
1	GO:0005515	Protein binding	8.15E−13	1.13E−09
2	GO:0044822	Poly(A) RNA binding	3.81E−07	5.29E−04
3	GO:0008134	Transcription factor binding	3.77E−06	5.23E−03
4	GO:0042802	Identical protein binding	1.11E−05	1.54E−02
5	GO:0031625	Ubiquitin protein ligase binding	2.41E−05	3.34E−02
	*Cellular component*			
1	GO:0005829	Cytosol	6.27E−12	8.32E−09
2	GO:0005654	Nucleoplasm	8.76E−08	1.16E−04
3	GO:0005741	Mitochondrial outer membrane	2.53E−06	3.36E−03
4	GO:0005634	Nucleus	1.73E−05	2.29E−02
5	GO:0016020	Membrane	2.49E−05	3.31E−02
6	GO:0005739	Mitochondrion	3.34E−05	4.43E−02

Identified using the online Database for Annotation, Visualization, and Integrated Discovery (DAVID) v6.8.

## Discussion

In this study, we aimed to identify the diagnostic biomarker potential of circulating miRNAs for PTSD using serum samples from Dutch military subjects. We further aimed to gain insights in the coexpression patterns of these miRNAs, their predicted gene targets, and underlying biological pathways. Our preliminary findings suggest that 1) certain miRNAs could potentially serve as individual biomarkers of susceptibility, and 2) the coexpression of a specific set of miRNAs could accurately distinguish between subjects with PTSD, resilient individuals, and nonexposed controls. Such markers could be useful in clinical settings for accurate diagnosis and treatment planning, which is especially relevant for individuals who have that have difficulties associating their symptoms to a traumatic event, are unable to describe their symptoms, or are unwilling to fully disclose them (Yehuda et al., 2013).

Differential expression analysis identified 1 differentially expressed miRNA between subjects with PTSD and resilient individuals and 22 between subjects with PTSD and nonexposed controls (after multiple testing correction and a log2 FC cutoff of ≥|1|). Of these, miR-138-5p was significantly overexpressed in subjects with PTSD as compared to controls, and WGCNA revealed that this was the hub miRNA of the blue module. Serum levels of this miRNA were previously found altered in a rat model of restraint stress (Balakathiresan et al., 2014), while hippocampal miR-138-5p levels were associated with the formation of fear memories in mice (Li et al., 2018). Another miRNA, miR-1246, was the only significant miRNA that was downregulated in PTSD cases compared to resilient subjects and had a log2 FC>|1|. This miRNA was previously found downregulated in peripheral blood mononuclear cells of war veterans suffering from PTSD as compared to healthy nontrauma-exposed controls (Bam et al., 2016b). Such findings suggest that these miRNAs could be implicated in PTSD and potentially aid in diagnosing this disorder.

The three modules of coexpressed miRNAs identified by WGCNA revealed that most of the detected miRNAs could be clustered based on similarities in their expression patterns. The blue module contained 79 miRNAs which could significantly differentiate between trauma-exposed individuals and nonexposed controls. Interestingly, within the trauma-exposed individuals, the expression profiles of these miRNAs were significantly different between individuals with and without PTSD. This highlights the importance of including and studying not only non-trauma exposed controls but also trauma-exposed healthy individuals in order to disentangle PTSD effects from trauma-related effects. Moreover, 67 of the miRNAs of the blue module, including its hub miRNA, were significantly differentially expressed between PTSD cases and controls, which enhances the notion that these miRNAs could be relevant for PTSD.

Of the five miRNAs with the highest module membership, we calculated the AUCs to assess their diagnostic accuracy (Grund and Sabin, 2010). In order to determine the biomarker potential of these miRNAs, i.e., their potential to reflect PTSD regardless of any other confounding condition, we used uncorrected miRNA expression values. Interestingly, the results suggest that these miRNAs could almost perfectly distinguish PTSD subjects from resilient individuals and controls. However, these results were not reflected by the DESeq2 analyses in which the expression levels of these miRNAs were not different between PTSD cases and resilient individuals. Part of this discrepancy can most likely be attributed to confounding effects, as DESeq2 analyses were corrected for age, alcohol, and smoking status. When obtaining the ROC curves using confounder-adjusted miRNA expression values, the AUCs more accurately corresponded to the DESeq2 results. Although these results suggest that the expression of our selected miRNAs fluctuates with confounders, they mostly strengthen the need of replication in larger cohorts. This will further be valuable in determining whether these miRNAs could be specific for PTSD only as opposed to trauma more broadly.

Enrichment of GO terms indicated that target genes of the coexpressed miRNAs in the blue module are enriched in several KEGG pathways of which most were cancer-related. This suggests that these miRNAs could be implicated in cancer pathways that are also involved in signaling cascades possibly related to PTSD. The target genes were also involved in several biological processes of which most were involved in apoptotic processes. Previous studies found reduced level of apoptotic markers in the serum of subjects with PTSD (Mkrtchian et al., 2013) and abnormal apoptosis in specific brain regions of animals undergoing single prolonged stress as a model for PTSD (Han et al., 2013; Li et al., 2013; Jia et al., 2018). These findings indicate a potential apoptosis dysfunction that could contribute to the inflammation pattern frequently observed within PTSD (Mkrtchian et al., 2013). Furthermore, the involvement of the identified genes in cellular responses after mechanical stimuli could indicate the need to correct for traumatic brain injuries, which are not uncommon among military members. Unfortunately, this information was not available for the present study. Finally, enriched molecular function GO terms suggest their involvement in the binding of proteins and RNA, while the significant cellular component GO terms show involvement of intracellular compartments such as the cytosol and the nucleoplasm.

Of note, the present paper refers to trauma-exposed healthy individuals as being “resilient” in order to create a clear differentiation between trauma-exposed healthy subjects and nonexposed control subjects. However, we do acknowledge and emphasize that resilience is more than just the reverse side of PTSD or the absence of symptomatology (Kalisch et al., 2017; Snijders et al., 2018b). Instead, resilience is an active and dynamic process that needs to remain separated from the multifaceted and complex nature of PTSD. This complexity further suggests that identifying one true and valid biomarker of susceptibility is likely not realistic. We therefore urge future studies to combine findings such as the ones presented in this paper with several other biological networks and phenotypic profiles in order to develop a cross-dimensional, global understanding of PTSD.

The main strength of this study lies in the inclusion of three different groups, i.e., PTSD subjects, resilient subjects, and nonexposed healthy controls, which allows us to disentangle PTSD- from trauma-related effects. However, the study is mainly limited by its relatively small sample size consisting of male subjects only. Given the existence of female- and male-biased miRNAs as recently reported by Cui, Yang et al. (2018) (Cui et al., 2018), these findings may not be applicable to the female population. This study population may also differ from other cohorts such as civilians in terms of demographics, psychological characteristics, and type of experienced trauma, which limits the extrapolation potential. Next, one could question the validity of self-report PTSD measures and whether the observed markers are specific to PTSD since certain comorbidities such as (history of) traumatic brain injuries were not available and thus not accounted for.

In conclusion, this paper presents preliminary evidence for using specific miRNAs as diagnostic biomarkers of PTSD, either individually or grouped within coexpressed clusters. Identifying reliable biomarkers of PTSD is essential for accurate diagnosis and treatment planning. We therefore encourage future studies to build upon these findings by aiming to replicate these in larger cohorts and thus pave the way for functional studies to gain insights into the precise roles of these miRNAs in stress susceptibility.

## Data Availability Statement

The Data is available at GEO, accession: GSE137624.

## Ethics Statement

The studies involving human participants were reviewed and approved by The ethical committee of University Medical Center Utrecht (01-333/0). The patients/participants provided their written informed consent to participate in this study.

## Author Contributions

LN, BR, GK, DH, JKl, and MK participated in the conception and the design of the study. MB, CV, EV, and EG recruited the participants and collected the blood samples. CS and BM performed the experiments. CS, JKr, EP, and LE performed the bioinformatics analysis. CS wrote the manuscript. LN, BR, and MK provided the necessary fundings. All authors critically read, commented, provided scientific content and approved the final manuscript.

## Funding

This work has been funded by the European Union’s Horizon 2020 research and innovation program under the Marie Sklodowska-Curie grant agreement N0. 707362 (LN) and by a VIDI award number 91718336 from the Netherlands Scientific Organization (BR).

## Conflict of Interest

The authors declare that the research was conducted in the absence of any commercial or financial relationships that could be construed as a potential conflict of interest.

## Supplementary Material

Supplementary Figure S1 Sample dendrogram to detect outliers. Clustering was based on miRNA expression data. Sample names were relabeled as 1-20.

Supplementary Figure S2 Correlations of modules detected by WGCNA and the following potential covariates: age, smoking status and alcohol use. P-values are presented between brackets. ME: module eigengene.

Supplementary Figure S3 Receiver operating characteristic (ROC) curve for the miRNA with the highest module membership in the blue module, i.e. miR-221-3p. The graphs represent PTSD vs control without confounders (A.1) or with confounders (B.1), and PTSD vs resilient without confounders (A.2) or with confounders (B.2).
