# Supplementary figures and images for: Circulating Serum MicroRNAs as Potential Diagnostic Biomarkers of Posttraumatic Stress Disorder: A Pilot Study
Source: Front Genet. 2019 Nov 22;10:1042. doi: 10.3389/fgene.2019.01042 (PMC6883918; doi:10.3389/fgene.2019.01042)

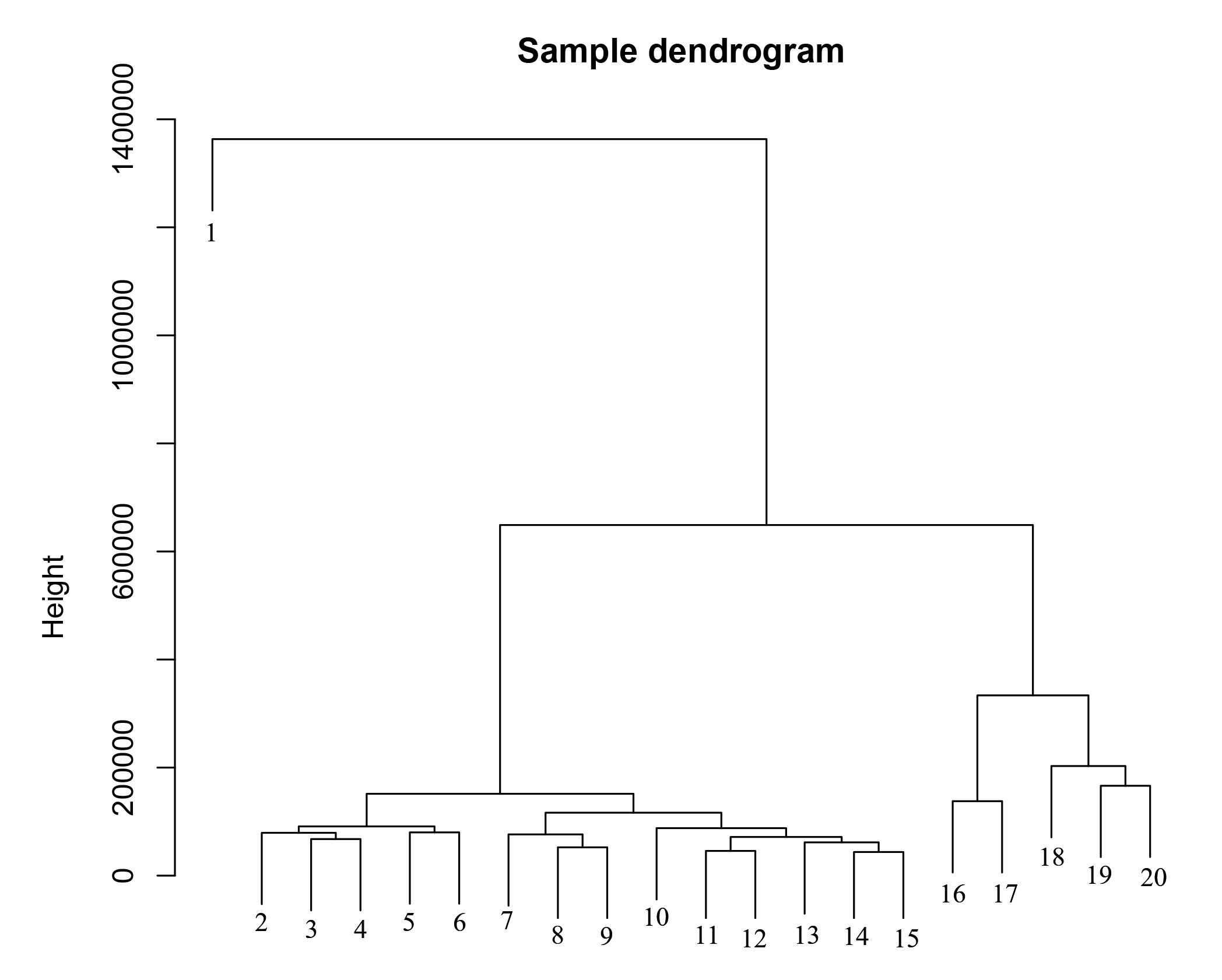

Supplement: Supplementary Figure S1 — Sample dendrogram to detect outliers. Clustering was based on miRNA expression data. Sample names were relabeled as 1-20. [file Image_1.tif]

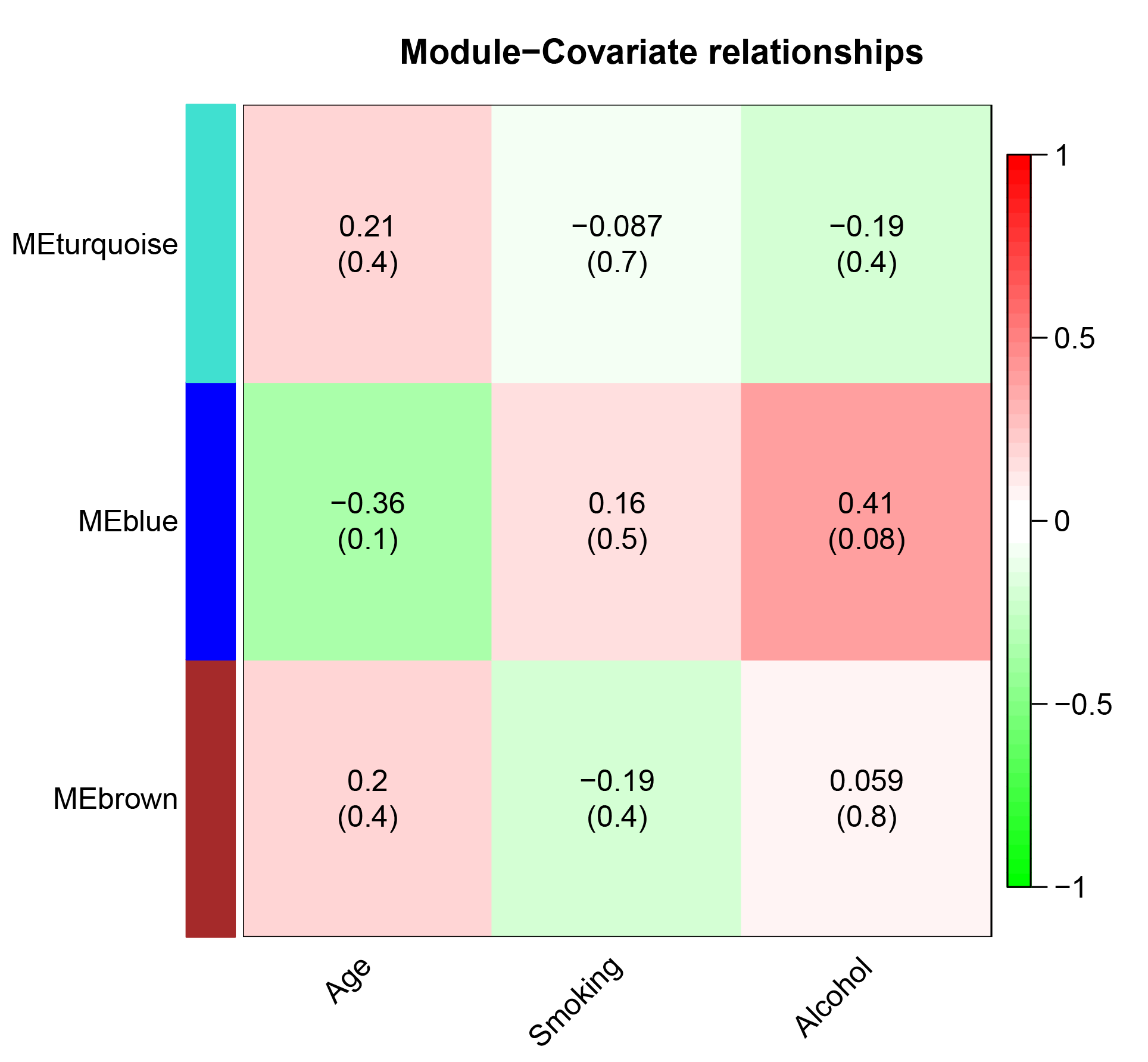

Supplement: Supplementary Figure S2 — Correlations of modules detected by WGCNA and the following potential covariates: age, smoking status and alcohol use. P-values are presented between brackets. ME: module eigengene. [file Image_2.tif]

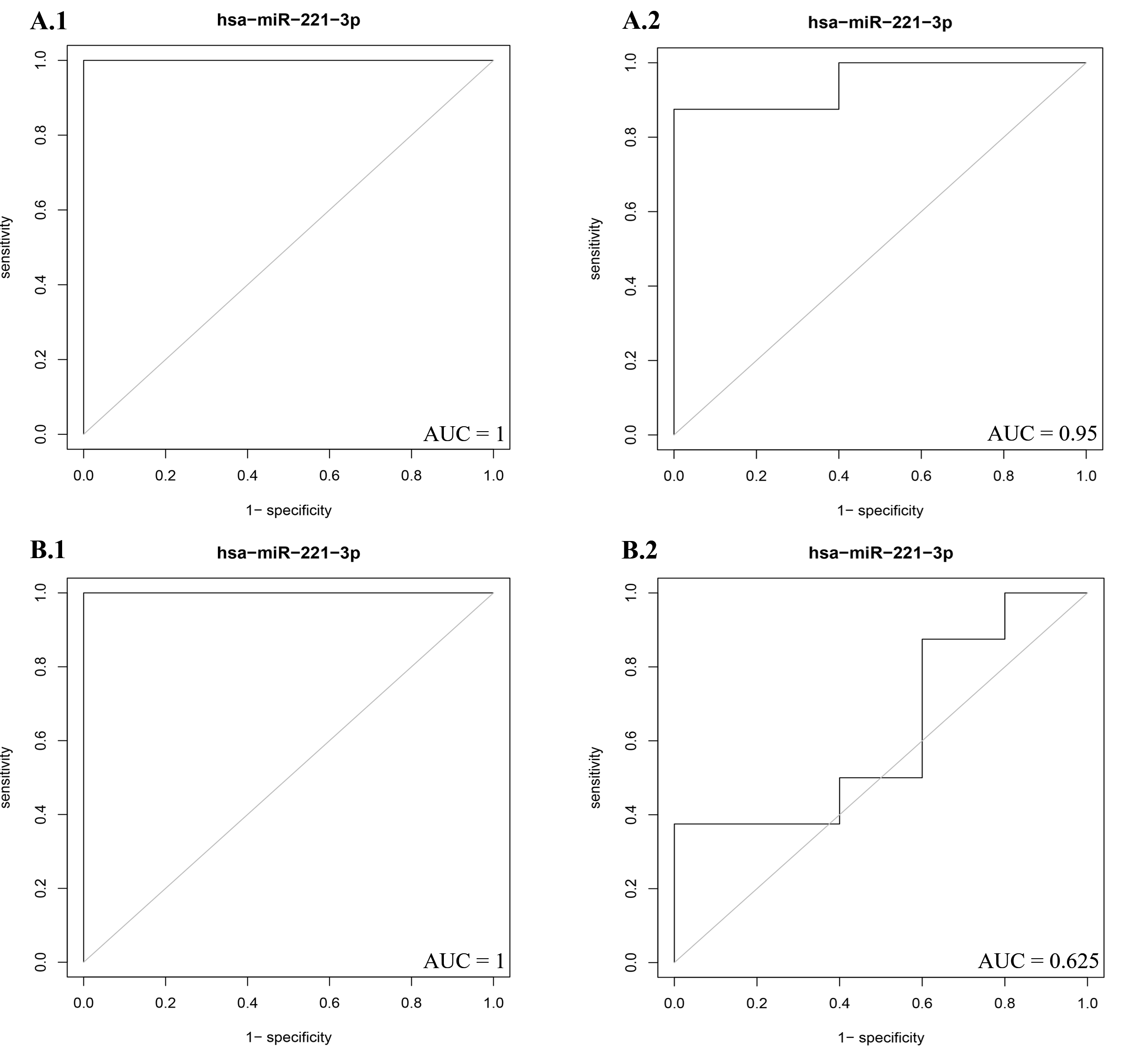

Supplement: Supplementary Figure S3 — Receiver operating characteristic (ROC) curve for the miRNA with the highest module membership in the blue module, i.e. miR-221-3p. The graphs represent PTSD vs control without confounders (A.1) or with confounders (B.1), and PTSD vs resilient without confounders (A.2) or with confounders (B.2). [file Image_3.tif]
