# Supplementary material for: Circulating Serum MicroRNAs as Potential Diagnostic Biomarkers of Posttraumatic Stress Disorder: A Pilot Study
Source: Front Genet. 2019 Nov 22;10:1042. doi: 10.3389/fgene.2019.01042 (PMC6883918; doi:10.3389/fgene.2019.01042)
Supplement: Supplementary file 4 [file DataSheet_1.zip › Suppl Table 1 and 2.docx]

**Circulating serum microRNAs as potential diagnostic biomarkers of post-traumatic stress disorder: A pilot study**

**Supplementary material**

Snijders, C., Krauskopf, J., Pishva, E., Eijssen, L., Machiels, B., Kleinjans, J., Kenis, G., van den Hove, D., Boks MPM, Vinkers, CH., Eric Vermetten, E., Geuze, E., Rutten, BPF., de Nijs, L.

| **Supplementary Table S1**. Demographic characteristics of 24 subjects before outlier exclusion. | | | | |
| --- | --- | --- | --- | --- |
|  | Susceptible  (N = 8) | Resilient  (N = 8) | Control  (N = 8) | *P*-value |
| Age when deployed | 22.13 (0.61) | 32.75 (4.03) | 26.75 (2.71) | .047 |
| Number of previous deployments | 0.29 (0.18) | 1.13 (0.55) | 0.25 (0.16) | .358 |
| Military rank | 1.38 (0.26) | 2.13 (0.40) | 1.5 (0.27) | .222 |
| Cigarette smoking at baseline | 2 (0.62) | 0.38 (0.26) | 0.38 (0.38) | .093 |
| Alcohol use | 2.86 (0.67) | 1.25 (0.31) | 1.88 (0.44) | .130 |
| Trauma exposure-score | 7.75 (0.98) | 7.75 (0.67) | 0.5 (0.05) | <.001 |
| SRIP PTSD score | 55.25 (4.00) | 25.38 (1.32) | 24.50 (1.17) | <.001 |
| Data is presented as mean (SE). SRIP: Self-Rating Inventory for Post-traumatic stress disorder. | | | | |

| **Supplementary Table S2**. Differentially expressed miRNAs between PTSD subjects vs controls and PTSD subjects vs resilient subjects with FDR adjusted *p*-values <0.05. | | | | | | | | |
| --- | --- | --- | --- | --- | --- | --- | --- | --- |
|  | **PTSD vs control** | | | **PTSD vs resilient** | | | | |
| *Nr* | *miRNA* | *Log2 FC* | P*-value* | *FDR adj*  P*-value* | *miRNA* | *Log2 FC* | P*-value* | *FDR adj*  P*-value* |
| 1 | hsa-miR-218-2-3p | 3.22 | 1.65E-02 | 4.20E-02 | hsa-miR-210-3p | -0.54 | 2.57E-04 | 3.05E-02 |
| 2 | hsa-miR-3609 | 3.04 | 8.22E-06 | 6.62E-05 | hsa-miR-4286 | -0.54 | 6.82E-04 | 4.40E-02 |
| 3 | hsa-miR-432-5p | 2.37 | 8.44E-04 | 3.31E-03 | hsa-miR-4454 | -0.61 | 3.94E-07 | 1.02E-04 |
| 4 | hsa-miR-138-5p | 2.29 | 2.18E-16 | 9.54E-15 | hsa-miR-1246 | -1.06 | 3.54E-04 | 3.05E-02 |
| 5 | hsa-miR-221-5p | 2.06 | 6.11E-13 | 1.34E-11 |  |  |  |  |
| 6 | hsa-miR-4485-3p | 1.98 | 3.45E-15 | 9.59E-14 |  |  |  |  |
| 7 | hsa-miR-31-5p | 1.92 | 2.16E-15 | 7.35E-14 |  |  |  |  |
| 8 | hsa-miR-146b-5p | 1.67 | 1.86E-25 | 2.85E-23 |  |  |  |  |
| 9 | hsa-miR-5096 | 1.62 | 1.81E-06 | 1.84E-05 |  |  |  |  |
| 10 | hsa-miR-222-3p | 1.56 | 2.81E-15 | 8.61E-14 |  |  |  |  |
| 11 | hsa-miR-1273g-3p | 1.55 | 7.23E-05 | 3.75E-04 |  |  |  |  |
| 12 | hsa-miR-302a-5p | 1.49 | 6.61E-09 | 1.06E-07 |  |  |  |  |
| 13 | hsa-miR-221-3p | 1.45 | 1.03E-14 | 2.62E-13 |  |  |  |  |
| 14 | hsa-miR-619-5p | 1.40 | 1.74E-04 | 8.31E-04 |  |  |  |  |
| 15 | hsa-miR-335-5p | 1.28 | 7.18E-28 | 2.20E-25 |  |  |  |  |
| 16 | hsa-miR-146b-3p | 1.25 | 6.30E-11 | 1.13E-09 |  |  |  |  |
| 17 | hsa-miR-3175 | 1.17 | 1.95E-04 | 9.20E-04 |  |  |  |  |
| 18 | hsa-miR-3656 | 1.00 | 1.07E-02 | 2.86E-02 |  |  |  |  |
| 19 | hsa-miR-20b-5p | 0.98 | 4.65E-08 | 5.93E-07 |  |  |  |  |
| 20 | hsa-miR-214-3p | 0.92 | 6.21E-05 | 3.27E-04 |  |  |  |  |
| 21 | hsa-miR-193a-5p | 0.91 | 3.41E-04 | 1.49E-03 |  |  |  |  |
| 22 | hsa-miR-335-3p | 0.91 | 1.39E-03 | 4.93E-03 |  |  |  |  |
| 23 | hsa-miR-208b-3p | 0.88 | 4.08E-12 | 8.32E-11 |  |  |  |  |
| 24 | hsa-miR-34a-5p | 0.86 | 6.19E-06 | 5.16E-05 |  |  |  |  |
| 25 | hsa-miR-18a-3p | 0.86 | 1.09E-04 | 5.37E-04 |  |  |  |  |
| 26 | hsa-miR-1246 | 0.81 | 1.31E-03 | 4.79E-03 |  |  |  |  |
| 27 | hsa-miR-127-3p | 0.80 | 1.82E-03 | 6.39E-03 |  |  |  |  |
| 28 | hsa-miR-29a-3p | 0.70 | 1.22E-05 | 8.13E-05 |  |  |  |  |
| 29 | hsa-miR-324-3p | 0.67 | 3.22E-03 | 1.06E-02 |  |  |  |  |
| 30 | hsa-miR-675-5p | 0.67 | 2.99E-04 | 1.33E-03 |  |  |  |  |
| 31 | hsa-miR-490-5p | 0.62 | 3.53E-04 | 1.52E-03 |  |  |  |  |
| 32 | hsa-miR-641 | 0.60 | 3.12E-03 | 1.04E-02 |  |  |  |  |
| 33 | hsa-miR-532-5p | 0.59 | 7.11E-18 | 4.35E-16 |  |  |  |  |
| 34 | hsa-miR-708-5p | 0.57 | 2.15E-03 | 7.48E-03 |  |  |  |  |
| 35 | hsa-miR-99b-3p | 0.56 | 1.15E-05 | 7.83E-05 |  |  |  |  |
| 36 | hsa-miR-199a-5p | 0.55 | 3.26E-03 | 1.06E-02 |  |  |  |  |
| 37 | hsa-miR-140-5p | 0.54 | 2.29E-03 | 7.87E-03 |  |  |  |  |
| 38 | hsa-miR-346 | 0.53 | 2.69E-03 | 9.06E-03 |  |  |  |  |
| 39 | hsa-miR-363-3p | 0.53 | 3.36E-06 | 2.94E-05 |  |  |  |  |
| 40 | hsa-miR-424-3p | 0.50 | 2.45E-05 | 1.47E-04 |  |  |  |  |
| 41 | hsa-miR-199a-3p | 0.48 | 5.00E-07 | 5.46E-06 |  |  |  |  |
| 42 | hsa-miR-199b-3p | 0.48 | 6.09E-04 | 2.52E-03 |  |  |  |  |
| 43 | hsa-miR-19b-3p | 0.47 | 1.61E-05 | 1.01E-04 |  |  |  |  |
| 44 | hsa-miR-128-3p | 0.47 | 9.46E-06 | 6.97E-05 |  |  |  |  |
| 45 | hsa-miR-132-3p | 0.47 | 1.02E-05 | 7.24E-05 |  |  |  |  |
| 46 | hsa-miR-212-3p | 0.46 | 3.63E-03 | 1.15E-02 |  |  |  |  |
| 47 | hsa-miR-1307-5p | 0.43 | 1.51E-02 | 3.88E-02 |  |  |  |  |
| 48 | hsa-miR-20a-5p | 0.42 | 8.46E-06 | 6.62E-05 |  |  |  |  |
| 49 | hsa-miR-183-5p | 0.41 | 1.38E-02 | 3.61E-02 |  |  |  |  |
| 50 | hsa-miR-574-3p | 0.40 | 5.24E-03 | 1.57E-02 |  |  |  |  |
| 51 | hsa-miR-17-5p/106a-5p | 0.40 | 4.45E-08 | 5.92E-07 |  |  |  |  |
| 52 | hsa-miR-19a-3p | 0.39 | 4.10E-03 | 1.25E-02 |  |  |  |  |
| 53 | hsa-miR-505-3p | 0.38 | 7.38E-03 | 2.13E-02 |  |  |  |  |
| 54 | hsa-miR-504-5p | 0.34 | 8.65E-03 | 2.43E-02 |  |  |  |  |
| 55 | hsa-miR-331-3p | 0.31 | 1.33E-03 | 4.79E-03 |  |  |  |  |
| 56 | hsa-miR-24-2-5p | 0.26 | 9.92E-03 | 2.69E-02 |  |  |  |  |
| 57 | hsa-let-7b-5p | 0.26 | 1.42E-02 | 3.68E-02 |  |  |  |  |
| 58 | hsa-miR-130a-3p | 0.22 | 5.57E-04 | 2.33E-03 |  |  |  |  |
| 59 | hsa-miR-23a-3p | 0.15 | 9.53E-04 | 3.69E-03 |  |  |  |  |
| 60 | hsa-miR-151a-3p | 0.15 | 9.62E-03 | 2.65E-02 |  |  |  |  |
| 61 | hsa-miR-28-5p | -0.20 | 1.33E-03 | 4.79E-03 |  |  |  |  |
| 62 | hsa-miR-181a-3p | -0.21 | 7.07E-03 | 2.06E-02 |  |  |  |  |
| 63 | hsa-miR-30c-1-3p | -0.22 | 1.66E-02 | 4.20E-02 |  |  |  |  |
| 64 | hsa-miR-23b-3p | -0.24 | 7.86E-04 | 3.18E-03 |  |  |  |  |
| 65 | hsa-miR-874-5p | -0.25 | 1.89E-02 | 4.69E-02 |  |  |  |  |
| 66 | hsa-miR-26a-5p | -0.25 | 5.23E-03 | 1.57E-02 |  |  |  |  |
| 67 | hsa-miR-454-5p | -0.27 | 8.19E-03 | 2.34E-02 |  |  |  |  |
| 68 | hsa-miR-675-3p | -0.29 | 4.05E-03 | 1.25E-02 |  |  |  |  |
| 69 | hsa-miR-652-3p | -0.30 | 8.87E-03 | 2.47E-02 |  |  |  |  |
| 70 | hsa-miR-210-5p | -0.32 | 1.84E-02 | 4.61E-02 |  |  |  |  |
| 71 | hsa-miR-361-3p | -0.33 | 4.62E-04 | 1.96E-03 |  |  |  |  |
| 72 | hsa-miR-181c-3p | -0.33 | 6.59E-03 | 1.94E-02 |  |  |  |  |
| 73 | hsa-miR-191-5p | -0.33 | 1.23E-02 | 3.26E-02 |  |  |  |  |
| 74 | hsa-miR-301b-3p | -0.36 | 3.41E-05 | 1.97E-04 |  |  |  |  |
| 75 | hsa-miR-1287-5p | -0.36 | 9.84E-03 | 2.69E-02 |  |  |  |  |
| 76 | hsa-miR-181a-2-3p | -0.36 | 6.24E-06 | 5.16E-05 |  |  |  |  |
| 77 | hsa-miR-340-5p | -0.37 | 3.35E-05 | 1.97E-04 |  |  |  |  |
| 78 | hsa-miR-143-5p | -0.38 | 1.18E-03 | 4.52E-03 |  |  |  |  |
| 79 | hsa-miR-378a-3p/378c/378d/378e | -0.39 | 3.45E-03 | 1.10E-02 |  |  |  |  |
| 80 | hsa-miR-30d-5p | -0.40 | 1.06E-05 | 7.38E-05 |  |  |  |  |
| 81 | hsa-miR-92b-5p | -0.42 | 8.63E-03 | 2.43E-02 |  |  |  |  |
| 82 | hsa-miR-27a-3p/27b-3p | -0.43 | 3.53E-07 | 4.15E-06 |  |  |  |  |
| 83 | hsa-miR-181b-5p | -0.44 | 8.66E-06 | 6.62E-05 |  |  |  |  |
| 84 | hsa-miR-2110 | -0.45 | 1.24E-02 | 3.26E-02 |  |  |  |  |
| 85 | hsa-miR-1180-3p | -0.45 | 5.28E-03 | 1.57E-02 |  |  |  |  |
| 86 | hsa-miR-342-3p | -0.45 | 2.58E-03 | 8.76E-03 |  |  |  |  |
| 87 | hsa-miR-125a-5p | -0.46 | 7.91E-04 | 3.18E-03 |  |  |  |  |
| 88 | hsa-miR-628-5p | -0.46 | 8.00E-04 | 3.18E-03 |  |  |  |  |
| 89 | hsa-miR-7706 | -0.46 | 8.18E-05 | 4.17E-04 |  |  |  |  |
| 90 | hsa-miR-130b-5p | -0.46 | 2.65E-06 | 2.46E-05 |  |  |  |  |
| 91 | hsa-let-7i-5p | -0.48 | 4.90E-07 | 5.46E-06 |  |  |  |  |
| 92 | hsa-miR-148b-5p | -0.49 | 1.26E-03 | 4.72E-03 |  |  |  |  |
| 93 | hsa-miR-143-3p | -0.49 | 1.65E-04 | 8.01E-04 |  |  |  |  |
| 94 | hsa-let-7a-5p/7c-5p | -0.51 | 6.86E-07 | 7.24E-06 |  |  |  |  |
| 95 | hsa-miR-148a-3p | -0.51 | 3.97E-05 | 2.21E-04 |  |  |  |  |
| 96 | hsa-miR-99a-5p | -0.54 | 2.52E-04 | 1.15E-03 |  |  |  |  |
| 97 | hsa-miR-181a-5p | -0.55 | 3.75E-05 | 2.13E-04 |  |  |  |  |
| 98 | hsa-miR-3200-3p | -0.56 | 4.85E-08 | 5.94E-07 |  |  |  |  |
| 99 | hsa-miR-208a-5p | -0.57 | 8.71E-05 | 4.37E-04 |  |  |  |  |
| 100 | hsa-miR-3605-3p | -0.58 | 1.73E-05 | 1.06E-04 |  |  |  |  |
| 101 | hsa-miR-455-5p | -0.60 | 2.65E-06 | 2.46E-05 |  |  |  |  |
| 102 | hsa-miR-181c-5p | -0.60 | 8.22E-09 | 1.26E-07 |  |  |  |  |
| 103 | hsa-miR-204-5p | -0.62 | 1.21E-03 | 4.57E-03 |  |  |  |  |
| 104 | hsa-miR-145-3p | -0.63 | 5.08E-05 | 2.73E-04 |  |  |  |  |
| 105 | hsa-let-7a-3p | -0.63 | 2.26E-04 | 1.05E-03 |  |  |  |  |
| 106 | hsa-miR-208a-3p | -0.64 | 3.40E-03 | 1.10E-02 |  |  |  |  |
| 107 | hsa-miR-628-3p | -0.66 | 2.32E-06 | 2.29E-05 |  |  |  |  |
| 108 | hsa-miR-148a-5p | -0.68 | 3.74E-03 | 1.17E-02 |  |  |  |  |
| 109 | hsa-miR-340-3p | -0.70 | 3.10E-08 | 4.31E-07 |  |  |  |  |
| 110 | hsa-miR-425-3p | -0.72 | 2.65E-04 | 1.19E-03 |  |  |  |  |
| 111 | hsa-let-7g-5p | -0.74 | 5.82E-13 | 1.34E-11 |  |  |  |  |
| 112 | hsa-miR-210-3p | -0.74 | 6.50E-10 | 1.10E-08 |  |  |  |  |
| 113 | hsa-let-7d-3p | -0.74 | 1.31E-05 | 8.54E-05 |  |  |  |  |
| 114 | hsa-let-7f-5p | -0.75 | 1.39E-16 | 7.09E-15 |  |  |  |  |
| 115 | hsa-miR-10a-5p | -0.75 | 9.56E-06 | 6.97E-05 |  |  |  |  |
| 116 | hsa-miR-4454 | -0.81 | 6.61E-16 | 2.53E-14 |  |  |  |  |
| 117 | hsa-miR-125b-2-3p | -0.82 | 8.74E-12 | 1.67E-10 |  |  |  |  |
| 118 | hsa-miR-4662a-5p | -0.85 | 2.84E-06 | 2.56E-05 |  |  |  |  |
| 119 | hsa-miR-1226-3p | -0.88 | 4.56E-05 | 2.49E-04 |  |  |  |  |
| 120 | hsa-miR-184 | -1.21 | 1.58E-05 | 1.01E-04 |  |  |  |  |
| 121 | hsa-let-7d-5p | -1.27 | 1.22E-18 | 9.33E-17 |  |  |  |  |
| 122 | hsa-miR-98-5p | -1.33 | 1.57E-22 | 1.60E-20 |  |  |  |  |
| 123 | hsa-miR-146a-5p | -2.04 | 1.52E-08 | 2.22E-07 |  |  |  |  |
| The table is organized based on decreasing log2 fold-change values. miRNA: microRNA, log2 FC: log2 fold-change. | | | | | | | | |
